# Confidentiality Issues and Use of Sexually Transmitted Disease Services Among Sexually Experienced Persons Aged 15–25 Years — United States, 2013–2015

**DOI:** 10.15585/mmwr.mm6609a1

**Published:** 2017-03-10

**Authors:** Jami S. Leichliter, Casey Copen, Patricia J. Dittus

**Affiliations:** ^1^Division of Sexually Transmitted Disease Prevention, National Center for HIV/AIDS, Viral Hepatitis, STD, and TB Prevention, CDC; ^2^Division of Vital Statistics, National Center for Health Statistics, CDC.

National-level data are limited regarding confidentiality-related issues and the use of sexually transmitted disease (STD) services for adolescents and young adults. Changes in the U.S. health care system have permitted dependent children to remain on a parent’s health insurance plan until the child’s 26th birthday and required coverage of certain preventive services, including some STD services, without cost sharing for most plans (*1*,*2*). Although these provisions likely facilitate access to the health care system, adolescents and young adults might not seek care or might delay seeking care for certain services because of concerns about confidentiality, including fears that their parents might find out (*3*,*4*). Therefore, it is important to examine STD services and confidentiality-related issues among persons aged 15–25 years in the United States. CDC analyzed data from the 2013–2015 National Survey of Family Growth and found that 12.7% of sexually experienced youths (adolescents aged 15–17 years and those young adults aged 18–25 years who were on a parent’s insurance plan) would not seek sexual and reproductive health care because of concerns that their parents might find out. Particularly concerned were persons aged 15–17 years (22.6%). Females with confidentiality concerns regarding seeking sexual and reproductive health care reported a lower prevalence of receipt of chlamydia screening (17.1%) than did females who did not cite such concerns (38.7%). More adolescents aged 15–17 years who spent time alone with a health care provider (without a parent in the room) reported receipt of a sexual risk assessment (71.1%) and, among females, chlamydia testing (34.0%), than did those who did not spend time alone (36.6% and 14.9%, respectively). The results indicated that confidentiality-related issues were associated with less reported use of some STD services, especially for younger persons and females. Spending time alone with a provider (i.e., without a parent present) during a health care visit has been associated previously with higher reported delivery of sexual health services (*5*) and has been suggested by the American Academy of Pediatrics and Society for Adolescent Health and Medicine (*6*). Public health efforts related to confidentiality of STD services might be helpful to increase the use of recommended services among some youths.

To effectively prevent and control the spread of STDs, CDC recommends health services that include a sexual risk assessment, chlamydia screening for sexually active women aged ≤25 years, and risk-based testing for other STDs (*7*). Several professional medical organizations have endorsed approaches to maintaining confidentiality in insurance plan communications (e.g., explanation of benefits) (*4*). This report uses data for sexually experienced persons aged 15–25 years to provide national estimates of confidentiality-related issues among U.S. adolescents and young adults and examines that association with the receipt of STD services.

The National Survey of Family Growth conducts in-person interviews with females and males aged 15–44 years selected from U.S. households and collects information on marriage, divorce, family life, having and raising children, and medical care.* The survey measures reproductive health status and evaluates the need for and effectiveness of health education programs. The 2013–2015 survey included 10,205 respondents with a 69.3% response rate.

For this report, the data used were primarily collected using audio computer-assisted self-interviewing. STDs are transmitted by sexual contact; therefore, analyses were restricted to respondents aged 15–25 years who were sexually experienced, defined as ever having had any type of sexual contact (vaginal, oral, or anal) with an opposite-sex or same-sex partner. Confidentiality-related issues in the survey included 1) whether all respondents aged 15–17 years and those respondents aged 18–25 years who were on a parent’s private health insurance plan would “ever not go for sexual or reproductive health care because their parents might find out”; 2) whether respondents aged 15–17 years had “time alone with a provider in the past 12 months without a parent, relative, or guardian in the room”; and 3) current health insurance status, including being on a parent’s insurance plan. STD services included receiving a sexual risk assessment and other clinical services. Receipt of a sexual risk assessment in the past 12 months was defined as reporting that a doctor or other health care provider asked about at least one of the following: 1) sexual orientation or sex of their sexual partners; 2) number of sexual partners; 3) use of condoms; and 4) types of sex (vaginal, oral, or anal). Receipt of other STD services was defined, for females, as receiving chlamydia testing in the past 12 months; for males, as receiving an STD test in the past 12 months; and for both females and males, as receiving treatment for an STD in the past 12 months.

Demographic characteristics of sexually experienced youths who would not seek sexual and reproductive health care because of concerns that their parents might find out were examined, and receipt of STD services was analyzed by demographic characteristics, sexual risk, and confidentiality-related issues. Analyses were weighted and adjusted to account for the complex survey design. Differences between groups were assessed using Wald chi-square tests, with statistical significance defined as p<0.05.

During 2013–2015, overall, 12.7% of sexually experienced persons aged 15–17 years and aged 18–25 years who were covered by a parent’s insurance plan (13.5% of females and 12.0% of males) reported that they would not seek sexual and reproductive health care because of concerns that their parents might find out (Table 1). A significantly higher percentage of youths aged 15–17 years (22.6%) said they would not seek sexual and reproductive health services for this reason than did those aged 20–22 years (8.2%) and 23–25 years (5.4%) (Table 1). Regarding receipt of STD services, persons aged 15–17 years who had time alone with a health care provider in the past 12 months reported significantly higher prevalences of receiving a sexual risk assessment (71.1%) than did those who did not have time alone with a provider (36.6%) (Table 2). Youths without health insurance reported the lowest prevalence of receiving a sexual risk assessment (38.2%), but the highest prevalence of receiving STD treatment (9.7%), compared with youths in other insurance categories.

**TABLE 1:** Percentage of sexually experienced* females and males aged 15–25 years who said they would not seek sexual or reproductive health care because their parents might find out,^†^ by demographic and behavioral characteristics — National Survey of Family Growth, United States, 2013–2015

Characteristic	Estimated pop.	% (95% CI)	p-value
**Total**	**17,077,000**	**12.7 (10.1–15.4)**	—
**Sex**			
Female	8,058,000	13.5 (10.1–16.9)	0.510
Male	9,019,000	12.0 (8.5–15.6)	
**Age group (yrs)**			
15–17	4,915,000	22.6 (17.6–27.6)	<0.001
18–19	3,013,000	14.1 (6.5–21.7)	
20–22	5,361,000	8.2 (4.2–12.2)	
23–25	3,789,000	5.4 (2.4–8.3)	
**Race/Ethnicity**			
Hispanic	2,985,000	14.7 (8.3–21.1)	0.161
White, non-Hispanic	10,746,000	12.1 (8.8–15.4)	
Black, non-Hispanic	2,115,000	9.9 (4.9–14.9)	
Other or multiple race, non-Hispanic	1,232,000	18.5 (8.0–28.9)	
**Composite sexual risk^§^**			
At elevated STD risk	1,981,000	17.1 (9.6–24.7)	0.225
Not at elevated STD risk	14,995,000	12.2 (9.4–15.0)	

**Abbreviations:** CI = confidence interval; STD = sexually transmitted disease.

* Sexually experienced was defined as those who have ever had vaginal intercourse, oral sex, or anal sex, with an opposite-sex or same-sex partner in their lifetime.

^†^ For respondents aged 18–25 years, this question was only asked if they were on a parent’s private health insurance plan.

^§^ Included male-to-male sex, females who had a male sex partner who had sex with other males, five or more sexual partners, sex in exchange for money or drugs, a sex partner who injected illegal drugs, or a human immunodeficiency virus–positive partner in the past 12 months.

**TABLE 2:** Percentage of sexually experienced* females and males aged 15–25 years who had received a selected STD-related service in the past 12 months, by confidentiality-related, sexual risk, and demographic characteristics — National Survey of Family Growth, United States, 2013–2015

Characteristic	Total				Females		Males	
	Sexual risk assessment % (95% CI)^†^	p-value	STD treatment % (95% CI)^§^	p-value	Chlamydia test % (95% CI)	p-value	STD test % (95% CI)^¶^	p-value
**Total**	**47.5 (44.8–50.3)**	**—**	**6.5 (5.3–7.6)**	**—**	**38.6 (35.9–41.2)**	**—**	**20.4 (17.5–23.2)**	**—**
**Confidentiality-related factors**								
Would ever not go for sexual or reproductive health care because their parents might find out**								
Yes	48.0 (39.6–56.4)	0.666	5.9 (1.3–10.5)	0.957	17.1 (6.6–27.7)	0.002	13.0 (4.4–21.6)	0.426
No	49.9 (46.1–53.7)		5.8 (3.8–7.7)		38.7 (34.0–43.4)		16.7 (13.0–20.4)	
**Had time alone with provider in past 12 months (15–17 yr age group only)**								
Yes	71.1 (62.8–79.3)	<0.001	6.6 (1.1–12.0)	0.072	34.0 (20.9–47.1)	0.021	13.6 (5.5–21.7)	0.424
No	36.6 (30.4–42.9)		1.4 (0.3–2.5)		14.9 (7.3–22.5)		9.5 (4.1–15.0)	
**Current health insurance**								
Private insurance, parent’s plan	49.3 (45.3–53.3)	<0.001	5.7 (3.8–7.6)	0.013	36.3 (30.9–41.6)	0.242	16.2 (12.1–20.3)	0.034
Private insurance, other	44.4 (37.1–51.6)		4.1 (2.2–6.1)		40.2 (29.0–51.4)		19.4 (11.5–27.3)	
Public insurance	51.9 (46.4–57.5)		7.2 (4.9–9.6)		43.4 (37.8–49.0)		24.9 (18.9–30.8)	
No insurance	38.2 (33.6–42.8)		9.7 (6.2–13.2)		35.4 (28.0–42.7)		24.7 (18.4–31.0)	
**Sexual risk**								
**Received sexual risk assessment in past 12 months^†^**								
Yes	—	—	10.9 (9.1–12.8)	<0.001	51.1 (47.1–55.0)	<0.001	42.9 (37.2–48.5)	<0.001
No	—	—	2.4 (1.2–3.6)		18.8 (14.2–23.3)		8.7 (6.3–11.2)	
**Composite sexual risk^††^**								
At elevated STD risk	60.6 (54.3–66.9)	0.001	19.6 (13.4–25.8)	<0.001	61.1 (50.8–71.3)	<0.001	44.4 (32.3–56.6)	0.001
Not at elevated STD risk	45.8 (42.6–49.0)		4.9 (3.8–5.9)		36.9 (33.9–39.7)		15.9 (13.4–18.3)	
**Demographics**								
**Age (yrs)**								
15–17	50.9 (45.5–56.4)	0.196	3.5 (1.1–5.9)	0.045	23.5 (16.5–30.4)	<0.001	10.7 (6.6–14.9)	0.002
18–19	51.3 (44.4–58.3)		7.6 (4.5–10.7)		31.4 (24.0–38.9)		15.4 (9.9–21.0)	
20–22	47.0 (42.5–51.5)		5.6 (3.9–7.4)		46.1 (40.7–51.6)		20.7 (16.2–25.2)	
23–25	44.9 (40.8–48.9)		8.0 (5.6–10.3)		40.6 (35.3–45.9)		27.4 (20.8–34.1)	
**Race/Ethnicity**								
Hispanic	49.1 (43.7–54.5)	0.001	5.6 (3.7–7.6)	<0.001	35.8 (28.8–42.7)	<0.001	23.9 (18.5–29.4)	<0.001
White, non-Hispanic	44.0 (40.0–48.0)		4.9 (3.7–6.2)		35.4 (31.3–39.5)		14.3 (11.1–17.5)	
Black, non-Hispanic	59.9 (54.9–64.8)		12.6 (9.4–15.9)		56.1 (49.5–62.7)		38.4 (30.3–46.4)	
Other or multiple race, non-Hispanic	43.6 (36.1–51.1)		7.2 (2.4–12.1)		35.1 (24.9–45.4)		15.8 (5.9–25.7)	

**Abbreviations:** CI = confidence interval; STD = sexually transmitted disease.

* Sexually experienced was defined as those who have ever had vaginal intercourse, oral sex, or anal sex, with an opposite-sex or same-sex partner in their lifetime.

^†^ Based on at least one “yes” response to four questions asking whether a doctor or other medical care provider asked about the sexual orientation or the sex of their sexual partners, number of sexual partners, use of condoms, and the types of sex they have (vaginal, oral, or anal).

^§^ “In the past 12 months, have you been treated or received medication from a doctor or other medical care provider for a sexually transmitted disease like gonorrhea, chlamydia, herpes, or syphilis?”

^¶^ “In the past 12 months, have you been tested by a doctor or other medical care provider for a sexually transmitted disease like gonorrhea, chlamydia, herpes, or syphilis?”

** For respondents aged 18–25 years, this question was only asked if they were on a parent’s private health insurance plan.

^††^ Included male-to-male sex, females who had a male sex partner who had sex with other males, five or more sexual partners, sex in exchange for money or drugs, a sex partner who injects illegal drugs, or a human immunodeficiency virus (HIV)–positive partner in the past 12 months.

Other recommended STD services also were examined by confidentiality-related issues. Significantly lower percentages of females who reported that they would not seek sexual and reproductive health care because of concerns that their parents might find out received a chlamydia test in the past 12 months (17.1%) than did those who did not report this concern (38.7%). In addition, females aged 15–17 years who had time alone with a health care provider were significantly more likely to have received a chlamydia test in the past 12 months (34.0%) than were those who had not had time alone with a provider (14.9%) (Table 2). Among males, the reported prevalence of receiving an STD test in the past 12 months did not differ significantly among those aged 15–25 years who would not go for sexual and reproductive health care because their parents might find out (13.0%) compared with those who would go (16.7%). The prevalence also did not differ significantly among males aged 15–17 years who had time alone with a provider in the past 12 months (13.6%) and those who did not (9.5%). Among males, the reported prevalences of receiving STD testing were significantly higher among those on public insurance (24.9%) and those with no insurance (24.7%) compared with those with private insurance (16.2%–19.4%).

## Discussion

Overall, 12.7% of sexually experienced persons aged 15–17 years and those aged 18–25 years on a parent’s insurance plan reported that they would not seek sexual and reproductive health care because of concerns that their parents might find out; these concerns were most commonly reported among persons aged 15–17 years (22.6%). Not seeking sexual and reproductive health care because of concerns that their parents might find out was associated with a lower prevalence of chlamydia testing among females. This finding is concerning because chlamydia is often asymptomatic, and chlamydia testing is a recommended preventive service for adolescent and young adult females (*7*). In addition, survey respondents who had time alone with their provider during their health care visit were more likely to have received a sexual risk assessment (both males and females) and a chlamydia test (females).

These findings are similar to those found for other sexual and reproductive health services (*8*). Several medical organizations have emphasized the need for confidentiality for youths seeking care such as STD services (*6*). Previous research has found that females might have more general and sexual and reproductive health–specific confidentiality concerns than do males (*9*). Finally, the frequency of STD testing among males with public insurance or no insurance was higher than among males with a parent’s insurance or private insurance. It is possible that these males might be seeking services from safety net providers (i.e., those who provide health care to uninsured or underinsured populations) who have reduced or no fees (*10*).

The findings in this report are subject to at least two limitations. First, receipt of STD services was self-reported and might be subject to social desirability and recall biases. Second, the survey was cross-sectional, and the confidentiality-related questions were not directly linked to the STD service questions. Thus, a causal relationship between confidentiality concerns and receipt of STD services cannot be inferred.

Concerns about maintaining confidentiality for STD services, including privacy issues such as not spending time alone with a health care provider without a parent in the room, might limit the use of these services for some youths. Public health practitioners might consider work to reduce some confidentiality concerns and potentially increase use of recommended STD services. Some medical organizations suggest that patients having time alone with a provider during a health care visit can be useful for sensitive services.

**Summary**

**What is already known about this topic?**

Issues related to confidentiality have been associated with youths not seeking care for some sexual or reproductive health–related services.

**What is added by this report?**

Nationally, 12.7% of sexually experienced adolescents and young adults who were on a parent’s health insurance plan would not seek sexual and reproductive health care because of concerns that their parents might find out. This was highest among persons aged 15–17 years (22.6%). Overall, these persons reported lower prevalences of receiving certain recommended sexually transmitted disease (STD) services. However, receiving a sexual risk assessment (both males and females) and chlamydia test (females) was higher among persons aged 15–17 years who had time alone with a health care provider in the past 12 months compared with those who had not.

**What are the implications for public health practice?**

Confidentiality issues, including concerns that parents might find out, might be barriers to the use of STD services among some subpopulations. Public health efforts to reduce these confidentiality concerns might be useful. Some medical organizations suggest that providers have time alone with patients without a parent in the room.

## Footnotes

* https://www.cdc.gov/nchs/nsfg/.
